# Clinical characteristics of COVID-19 patients admitted at the Korle-Bu Teaching Hospital, Accra, Ghana

**DOI:** 10.4314/gmj.v54i4s.6

**Published:** 2020-12

**Authors:** Patrick Adjei, Jane Afriyie-Mensah, Vincent J Ganu, Peter Puplampu, Bismark Opoku-Asare, Klenam Dzefi-Tettey, Maame-Boatemaa Amissah-Arthur, Kenneth Tachi, Vincent Boima, Dzifa Dey, Joseph Akamah, Albert Akpalu, Josephine Akpalu, Phillip Amoo, Elom Otchi, Kissinger Marfoh, Adwoa Agyei-Nkansah

**Affiliations:** 1 Department of Medicine and Therapeutics, University of Ghana Medical School, University of Ghana, Accra, Ghana; 2 Department of Medicine, Korle Bu Teaching Hospital, Accra, Ghana; 3 Department of Radiology, Korle Bu Teaching Hospital, Accra, Ghana; 4 Public Health Unit, Korle Bu Teaching Hospital, Accra, Ghana

**Keywords:** COVID-19, hospitalized, symptoms, complications, screening, pneumonia

## Abstract

The study examined the clinical characteristics and outcomes of 2019 novel coronavirus disease (COVID-19) infections among hospitalized patients.

**Design:**

Study design was a retrospective single-center review of hospital data.

**Setting:**

The study was conducted at the COVID-19 Treatment Center of the Department of Medicine and Therapeutics of the Korle-Bu Teaching hospital in Accra, Ghana.

**Participants and study tools:**

A total of fifty patients with laboratory (rRT-PCR) confirmed COVID-19 infection were involved in the study. A chart review of the medical records of the patients was conducted and the data obtained was documented using a data extraction form.

**Results:**

The median age was 53 years and most (36% (18/50)) of the patients were at least 60 years of age. Eighty percent (40/50) of the patients were symptomatic, with cough and difficulty in breathing being the commonest presenting symptoms. The mean duration of hospitalization was 12.3 ± 7.3 days. Hypertension and Diabetes Mellitus were the commonest co-morbidities occurring in 52% (26/50) and 42% (21/50) of patients respectively. Fifty percent of patients developed COVID-19 pneumonia as a complication. The mortality rate was 12% (6/50).

**Conclusion:**

In this study, SARS-CoV2 infection affected older adults with hypertension and diabetes mellitus being the common comorbidities. Patients with these comorbid conditions should be counselled by their clinicians to strictly observe the COVID-19 prevention protocols to reduce their risk of acquiring the infection. There is a need to pay critical and prompt attention to the management of patients with COVID-19 pneumonia particularly among people with diabetes to improve outcomes.

**Funding:**

None declared

## Introduction

The 2019 novel coronavirus disease (COVID-19) is a communicable respiratory disease caused by a novel strain of coronavirus suspected to be of zoonotic origin that causes illness in human beings.^1^,^2^ The novel coronavirus infection was first reported by China on 29^th^ December 2019 and rapidly evolved into a pandemic affecting many countries. The disease is spread from person to person through infected droplets from sneezing, coughing or talking.

Transmission can also be through physical contact of hands with infected surfaces and touching of face with contaminated hands. Transmission from asymptomatic contacts has been reported.^3^,^4^

The novel coronavirus pandemic moved swiftly from China and Asia to Europe and the Americas before Africa started experiencing its initial cases.

The first case of COVID-19 infection in Africa was reported in Egypt^5^ in February 2020 with Nigeria reporting the first COVID-19 case in Sub-Saharan Africa.^6^

As at 19^th^ July 2020, the total number of confirmed cases worldwide was over 14 million with over 600,000 deaths.^7^ Of these, Africa had over 700,000 cases with almost 15,000 deaths. South Africa had the highest of number of cases (350,879) and deaths (4948) in Africa with Ghana being the third country with the highest number of confirmed cases (27060) with 145 deaths.^7^

Although countries in Africa including Ghana are publicly reporting overall COVID-19 infection numbers and deaths regularly, there is little or no information on the clinical information of patients infected with COVID-19 infection in Ghana. Data on the epidemiological, clinical and laboratory characteristics of patients with COVID-19 infection have been reported in studies conducted in Asia (mainly China) with few from Europe.^8^,^9^,^18^–^20^,^10^–^17^

There is little of such information from countries in Africa including Ghana since the first recorded cases in Africa. Analysis of such data is required to inform health managers and service providers of early findings amongst our patients, which is needed for targeted management, prevention and health education approaches.

This study sought to determine the clinical characteristics, baseline co-morbidities and clinical outcomes of COVID-19 infected patients in Ghana.

## Methods

A retrospective single-centre review of hospital data was conducted among patients with laboratory confirmed diagnosis of SARS-CoV-2 infection and admitted to the COVID Treatment Center at the Medical Department of the Korle-Bu Teaching hospital from 16^th^ April to 30^th^ June 2020. The laboratory confirmation of the SARSCoV-2 infection amongst these patients were done using 2019-nCoV rRT-PCR at the Noguchi Memorial Institute for Medical Research (NMIMR) in Accra Ghana. Samples were obtained using nasopharyngeal swabs according to the recommended protocol by the Ghana National Covid-19 Guidelines.^21^.

Any patient with confirmed SARS-CoV-2 infection presenting with no clinical symptoms and signs from date of admission to discharge from hospital was classified as an asymptomatic patient. Patients with confirmed SARSCoV-2 infection with any clinical symptom or sign from date of admission to discharge from hospital was classified as a symptomatic patient.

A chart review of the medical records of the patients was conducted. Data was obtained from the medical records using a data extraction form. Data extracted from the medical files of patients included patients' socio-demographic characteristics and clinical characteristics.

The sociodemographic characteristics extracted included age, sex and occupation. Data on clinical characteristics extracted included clinical history, co-morbidities, duration of admission, complications developed during admission and clinical outcomes. Complications were described as Covid Pneumonia on Chest CT scan, Acute Kidney Injury (AKI), Deep Vein Thrombosis (DVT), Pleural Effusion whilst clinical outcomes were categorized into discharge from hospital and death from SARSCOV2 or its complications. The data extracted were reviewed by 2 independent research investigators as part of data validation and quality checks.

The study was conducted in accordance with the principles of Helsinki Declaration on Ethical Principles in human subject research. The Scientific and Technical Committee (STC) and the Institutional Review Board (IRB) of the Korle-Bu Teaching hospital approved this study with study ID KBTH STC/IRB 00095/2020.

### Statistical analysis

The data was entered into an excel sheet and exported into STATA version 13 for analysis. All categorical data were analyzed and presented as frequencies and percentages. The categorical data were compared using the chi square test or, if the data had less than ten expected frequencies, the Fischer's exact test was used. The chi square test was used to compare clinical symptoms to clinical outcomes and also to compare the effects of having a co-morbidity and the clinical outcome. Continuous variables were analyzed and presented as means with standard deviations, or median with quartile ranges. Significance level for all statistical analysis was set at a pvalue less than 0.05.

## Results

A total of 50 patients diagnosed with COVID-19 were admitted to the hospital of which 50% were female. The median age was 53 years with an age range of 9–86 years. Majority (36% (18/50)) of the patients were at least 60 years of age (Table 1). More than half (64% (32/50)) of the patients developed complications during admission.

**Table 1:** Demographic and clinical characteristics of hospitalized patients with COVID-19 at the Korle-Bu Teaching hospital, April - June 2020

Item	Frequency (%)
**Age**	
**Median (LQ, UQ)**	53 (36, 65)
**<25**	3 (6%)
**25–34**	9 (18%)
**35–44**	6 (12%)
**45–59**	14 (28%)
**≥60**	18 (36%)
**Sex**	
**Female**	25 (50%)
**Male**	25 (50%)
**Symptoms**	
**Asymptomatic**	10 (20%)
**Symptomatic**	40 (80%)
**Number of Symptoms**	
**0**	10 (20%)
**1**	11 (22%)
**≥2**	29 (58%)
**Complication**	
**No**	18 (36%)
**Yes**	32 (64%)
**Number of Complications**	
**1**	30 (93.7%)
**2**	2 (6.3%)
Types of complications *multiple **responses**	
**COVID-19 Pneumonia**	27 (84.4%)
**Acute Kidney Injury (AKI)**	5 (15.6%)
**Deep Vein Thrombosis (DVT)**	1 (3.1%)
**Pleural Effusion**	1 (3.1%)
**Co-morbidity**	
**No**	12 (24%)
**Yes**	38 (76%)
**Outcome**	
**Discharged**	44 (88%)
**Died**	6 (12%)

UQ=upper quadrant, LQ=lower quadrant, AKI=acute kidney injury, DVT=deep vein thrombosis

Twenty percent (10/50) of patients were asymptomatic. For those who were symptomatic, cough (57.5% (23/40)) and difficulty in breathing (52.5% (21/40), fever (45.0%, 18/40) and general malaise (20%) were the commonest presenting symptoms in the patients. Other complaints (23%) were headache, chest pain, diarrhea, confusion, chills, anorexia, abdominal pain, sore throat and anosmia.

### Duration of Hospitalization and Co-morbidities

The mean duration of hospitalization/admission was 12.3 ± 7.3 days with a range of 1 to 32 days. Twenty four percent (12/50) of the patients did not have any comorbidity and hypertension was the commonest co-morbidity occurring in 52% (26/50) of patients (Table 2).

**Table 2:** Co-morbidities among study participants (multiple responses)

Comorbid condition	N (%)
None	12 (24%)
Hypertension	26 (52%)
Diabetes Mellitus	21 (42%)
Kidney Disease	8 (16%)
CVD	7 (14%)
Asthma	3 (6%)
Retroviral Infection	3 (6%)
Others	5 (10%)

The other conditions comprised allergic rhinitis, post thyroidectomy left vocal cord paralysis, bleeding hemorrhoids, prostate cancer and deep vein thrombosis.

### Comorbid Condition, Symptoms at admission and the Development of Complications

Patients who had diabetes mellitus and hypertension showed statistically significant associations to developing complications (p =0.001) and (p = 0.01) respectively. Patients who showed symptoms at the time of admission were also more likely to have complications (p=0.001) (Table 3).

**Table 3:** Presence of Co-morbid Condition, symptoms at admission and the development of complications among study participants

	Complication		
CO-MORBIDITY	No (%)	Yes (%)	P-value
**Diabetes Mellitus**			
**No**	16(55.17)	13(44.83)	
**Yes**	2(9.52)	19(90.48)	0.001
**Hypertension**			
**No**	13(54.17)	11(45.83)	
**Yes**	5(19.23)	21(80.77)	0.01
**Asthma**			
**No**	16(34.04)	31(65.96)	
**Yes**	2(66.67)	1(33.33)	0.291
**Cardiovascular disease**			
**No**	17(39.53)	26(60.47)	
**Yes**	1(14.29)	6(85.71)	0.398
**Kidney disease**			
**No**	16(38.1)	26(61.9)	
**Yes**	2(25)	6(75)	0.694
**HIV Infection**			
**No**	18(38.3)	29(61.7)	
**Yes**	0(0)	3(100)	0.544
**SYMPTOMS**			
**Asymptomatic**	9(100)	0(0)	
**Symptomatic**	9(21.95)	32(78.05)	<0.001
**Number of Symptoms**			
**0**	9(100)	0(0)	
**1**	5(38.46)	8(61.54)	
**≥2**	4(14.29)	24(85.71)	<0.001

### Clinical Outcomes

The mortality rate among the number of patients admitted and managed for COVID-19 was 12% (6/50). There was no significant association between age and clinical outcomes (p = 0.95). No significant association between sex and clinical outcomes (p = 0.19) was observed (Table 3). All 6 patients who died had at least one co-morbidity. Five of them had both hypertension and diabetes mellitus whilst one had HIV infection. There was no statistically significant association between having any co-morbidity and the clinical outcome (See Table 4).

**Table 4:** COVID-19 complications, Comorbid Condition, Age, Gender and the clinical outcomes among study participants

	Outcome		
	Died	Discharged	P-value
**Age**			0.953
**<25**	0 (0)	3 (100)	
**25–34**	1 (11.11)	8 (88.89)	
**35–44**	0 (0)	6 (100)	
**45–59**	2 (14.29)	12 (85.71)	
**≥60**	3 (16.67)	15 (83.33)	
**Sex**			0.189
**Female**	5 (20)	20 (80)	
**Male**	1 (4)	24 (96)	
**Co-Morbidity**			
**Diabetes Mellitus**			
**No**	1 (3.45)	28 (96.55)	
**Yes**	5 (23.81)	16 (76.19)	0.07
**Hypertension**			
**No**	1 (4.17)	23 (95.83)	
**Yes**	5 (19.23)	21 (80.77)	0.192
**Asthma**			
**No**	6 (12.77)	41 (87.23)	
**Yes**	0 (0)	3 (100)	1
*Cardiovascular disease			
**No**	5 (11.63)	38 (88.37)	
**Yes**	1 (14.29)	6 (85.71)	1
**Chronic Kidney Disease**			
**No**	5 (11.9)	37 (88.1)	
**Yes**	1 (12.5)	7 (87.5)	1
**HIV Infection**			
**No**	5 (10.64)	42 (89.36)	
**Yes**	1 (33.33)	2 (66.67)	0.324
**Symptoms**			
**Cough**			
**No**	4 (14.81)	23 (85.19)	
**Yes**	2 (8.7)	21 (91.3)	0.674
**Difficulty in Breathing**			
**No**	2 (6.9)	27 (93.1)	
**Yes**	4 (19.05)	17 (80.95)	0.223
**Fever**			
**No**	2 (6.25)	30 (93.75)	
**Yes**	4 (22.22)	14 (77.78)	0.171
**General Malaise**			
**No**	4 (9.52)	38 (90.48)	
**Yes**	2 (25)	6 (75)	0.242
**Number of Symptoms**			
**0**	0 (0)	9 (100)	
**1**	0 (0)	13 (100)	
**≥2**	6 (21.43)	22 (78.57)	0.112
**Complication**			
**No**	0 (0)	18 (100)	
**Yes**	6 (18.75)	26 (81.25)	0.071

* Cardiovascular disease: dyslipidemia, ischemic stroke, congestive cardiac failure

## Discussion

The study sought to describe the preliminary clinical findings from management of patients with COVID-19 at a tertiary hospital in Ghana. Our early findings showed that majority of our patients were at least 60 years old.

Eighty percent of patients presented with symptoms with cough being the commonest. More than half of the patients had at least 2 symptoms.

Sixty-four percent of the patients developed complications with the commonest complication being Covid pneumonia. Seventy-five percent had co-morbidities with hypertension being the commonest co-morbidity and the mortality rate was 12%.

The median age in our study was similar to findings in other studies including a meta-analysis study in China^12^,^13^,^15^ but lower compared to findings from another study in Italy 14 and higher than a study in Saudi Arabia.^11^ This may be explained by the fact that adults may be more susceptible to SARS-COV2 infection and subsequent hospitalization as against the younger age group.^22^ However, the spike in the younger age group for the female gender may have been introduced by the small sample size with its attendant low power which may have increased the risk of a negative bias. There was equal distribution of infection with regards to gender which was contrary to reports from other studies where males were found to be more affected than females.^8^–^11^ The commonest symptom in our patients was cough which was corroborated by reports from previous studies.^10^,^11^,^19^ Contrary to other studies, fever was the third commonest symptoms amongst our patients.^8^,^9^,^23^

Hypertension was the commonest co-morbidity in our patients followed by diabetes mellitus. This is consistent with findings in other studies including one among an African American population where hypertension was reported as the commonest co-morbidity.^8^,^10^,^11^,^15^,^19^,^23^ This finding may also be a reflection of the high prevalence of hypertension and diabetes mellitus in the Ghanaian adult population.^24^,^25^ Similar to the study by Koh et al, Diabetes mellitus (DM) was the second co-morbidity in our study. Chronic Kidney disease was the third commonest comorbid condition, in contrast to findings by Koh et al who reported cardiovascular disease as the third commonest comorbid condition.^13^ The high prevalence of hypertension and diabetes mellitus in Ghana, the negative effects of these two conditions in the development of chronic kidney disease, as well as, the inadequate resources in managing end stage renal disease leads to an apparent high prevalence of CKD^26^,^27^ and may account for the observations in the study. Acute kidney injury has been reported in some studies^13^,^19^,^20^ as a complication of the novel coronavirus infection and similar findings were observed in this study.

Approximately 84% of the patients in our study developed COVID-19 pneumonia as a complication similar to findings from another study in hospitalized African American population.^19^ Other studies in non-African population also reported interstitial pneumonia as the commonest complication among patients with COVID-19.^9^,^14^,^23^

There was no significant difference in the distribution of complications between males and females in this study. Development of a complication was found to be closely linked to diabetes mellitus, hypertension and being symptomatic with more than two symptoms at the time of admission. This may be due to the decreased efficiency of both humoral and cellular immunity in diabetes mellitus^28^. Also, elevated levels of cytokines, visceral obesity and state of chronic inflammation in diabetes mellitus may also play a role in the development of complication.^29^,^30^

The mortality rate in this study was 12% with half of them being at least 60 years of age. There was no significant association between the clinical outcome and demographic and clinical characteristics. However, having diabetes mellitus and complications was associated with increasing mortality even though this did not have any statistical significance. In the setting of a low powered study such as the present study, increasing the power may support the risk of poor outcomes with the presence of comorbid conditions particularly diabetes mellitus. It is plausible that as diabetes mellitus affects both humoral and T-cell mediated immunity and is a hyper-coagulable state^29^,^30^, the presence of diabetes mellitus may carry a higher risk of poor outcomes in COVID-19 disease. In addition, T-cell mediated immunity that is typically compromised in HIV infection may have accounted for the lower incidence of poor outcomes in this group compared to other co-morbidities despite the high incidence of opportunistic infections and high mortality of HIV infections.

The limitation of this study is the small sample size. The sample size is small as this study sought to provide preliminary findings on patients managed in our setting. The small sample size is likely to reduce the power of the study and lead to higher variability.

## Conclusion

In this study, SARS-CoV2 infection affected older adults with hypertension and diabetes mellitus being the common comorbidities. Patients with these comorbid conditions should be counselled by their clinicians to strictly observe the COVID-19 prevention protocols to reduce their risk of acquiring the infection. There is a need to pay critical and prompt attention to the management of patients with COVID-19 pneumonia particularly among people with diabetes to improve outcomes.
